# Survival Analysis, Clinical Characteristics, and Predictors of Cerebral Metastases in Patients with Colorectal Cancer

**DOI:** 10.3390/medsci12030047

**Published:** 2024-09-02

**Authors:** Antoine Jeri-Yabar, Liliana Vittini-Hernandez, Jerry K. Benites-Meza, Sebastian Prado-Nuñez

**Affiliations:** 1 Department of Medicine, Icahn School of Medicine at Mount Sinai Morningside/West, New York, NY 10029, USA; liliana.vittinihernandez@mountsinai.org; 2 Sociedad Científica de Estudiantes de Medicina, Universidad Nacional de Trujillo, Trujillo 13001, Peru; jbenitesm@unitru.edu.pe; 3 Grupo Peruano de Investigación Epidemiológica, Unidad de Investigación para la Generación y Síntesis de Evidencias en Salud, Universidad San Ignacio de Loyola, Lima 15012, Peru; 4 Department of Medicine, Universidad Peruana Cayetano Heredia, Lima 15102, Peru; sebastianpradon@gmail.com

**Keywords:** colorectal cancer, brain metastases, survival analysis

## Abstract

Introduction: Colorectal cancer (CRC) is the third most common cancer globally and a leading cause of cancer-related deaths. While liver metastasis is common, brain metastasis (BM) is rare, occurring in 0.1% to 14% of cases. Risk factors for BM include lung metastasis at diagnosis, rectal cancer, and mutations in RAS and KRAS genes. Due to its rarity, guidelines for BM screening and treatment are limited. The aim of this study is to identify the clinical characteristics and predictors of BM at the time of the initial diagnosis of CRC. Methods: We evaluated patients ≥18 years old with metastatic colorectal cancer and brain metastases at diagnosis from the SEER database (2010–2021). A retrospective cohort study was conducted to analyze overall survival and predictive factors for brain metastasis, utilizing multivariate logistic regression, Kaplan–Meier survival analysis, and the Cox proportional hazards models, with *p*-values < 0.05 considered significant. Results: Out of 24,703 patients with metastatic colorectal cancer (mCRC), 228 (0.92%) had brain metastasis (BM) at diagnosis. BM was more prevalent in average-onset mCRC (≥50 years) compared to early-onset (<50 years) (1% vs. 0.55%, *p* = 0.004). Certain factors, such as older age and adenocarcinoma subtype, were associated with BM. Additionally, Asians/Pacific-Islanders (HR 1.83 CI: 1.01-3-33, *p* = 0.045) and American Indians/Alaska Natives (HR 4.79 CI 1.15–19.97, *p* = 0.032) had higher mortality rates, while surgical treatment and chemotherapy were linked to decreased mortality. Patients with BM had significantly worse overall survival (6 months vs. 21 months, *p* < 0.001). Conclusion: BM in mCRC is uncommon, but it is associated with significantly worse outcomes, including markedly reduced overall survival. Our study highlights several critical factors associated with the presence of BM, such as older age and specific racial/ethnic groups, which may inform risk stratification and early-detection strategies. Our findings emphasize the need for heightened awareness and screening for BM in high-risk mCRC patients, as well as the inclusion of these patients in clinical trials to explore tailored therapeutic approaches aimed at improving survival and quality of life.

## 1. Introduction

Colorectal cancer (CRC) is the third most common cancer worldwide and one of the leading causes of cancer-related deaths [1]. The liver is the most common site for metastasis in CRC, while brain metastasis (BM) is relatively rare, with prevalence rates ranging from 0.1% to 14% [2,3]. The presence of lung metastasis at the time of CRC diagnosis and rectal cancer are associated with a higher risk of developing BM [4]. Additionally, genetic mutations in the RAS and KRAS genes have been linked to an increased risk of BM in CRC patients [5]. 

Due to the rarity of BM from CRC, evidence and guidelines for screening and treatment are limited. It has been shown that around 96% of CRC patients with BM are asymptomatic [6]. The efficacy of chemotherapy is poor because the blood–brain barrier blocks the passage of cytotoxic drugs [7]. However, local treatment options like stereotactic radiosurgery (SRS) have become primary treatments for patients with limited or multiple BM [8]. Survival rates for CRC patients with BM are generally low, varying depending on the treatment approach. Patients receiving only supportive care have a median survival of 0.4 to 2 months, while those undergoing comprehensive therapeutic measures have a survival range of 12 to 41 months [7]. 

In recent years, advancements in cancer treatment, prognostic techniques, and systemic therapy have been significantly influenced by the development of immune checkpoint inhibitors. These inhibitors, particularly PD-1 inhibitors [9], have demonstrated promising results in treating brain metastases by stimulating a systemic immune response against malignant cells and crossing the blood–brain barrier. As a result, they have contributed to improved survival rates and a better quality of life for patients. Therefore, early diagnosis of brain metastasis is crucial to initiate prompt and effective treatment [10]. 

Given the challenges of treating BM and the often asymptomatic nature of BM in CRC patients, early detection is critical. However, direct screening for BM in all CRC patients is not feasible due to its low incidence. Therefore, identifying specific clinical characteristics and predictors of BM at the time of the initial diagnosis of CRC is vital. These predictors can guide targeted screening and more personalized treatment approaches, potentially improving survival rates and quality of life for affected patients.

The aim of this study is to identify the clinical characteristics and predictors of brain metastasis at the time of the initial diagnosis of CRC. Additionally, the study aims to describe overall survival over the past decade and the factors associated with it.

## 2. Methods

### 2.1. Population

Our study included individuals who were ≥18 years old and diagnosed with mCRC, who were then divided into two groups: patients with BM at diagnosis and those without BM at diagnosis. The data were acquired from the Surveillance, Epidemiology and End Results (SEER) database with a study period ranging from 2010 to 2021. The SEER database includes data on cancer incidence, survival, extent of disease, and treatment for 30% of the United States population. SEER has collected information regarding sites of metastases since 2010, hence the study period range that was chosen for this study. The study population included 24,703 patients. The groups were divided into patients with brain metastasis at diagnosis (*n* = 228) and other metastasis at diagnosis (*n* = 24,475). Inclusion factors included histologically confirmed CRC diagnosis based on the ICD-O-3 site codes, metastasis to other sites, known cause of death, confirmed metastatic colorectal cancer diagnosis, and first and only malignancy.

### 2.2. Study Design and Primary Outcome

Our study was a retrospective cohort study of survival analysis. The primary outcome was overall survival, which was defined as the time from cancer diagnosis to death. Secondary outcomes included predictive factors for brain metastasis at diagnosis of metastatic CRC. Study variables included age at diagnosis, sex, race, histologic subtype, tumor size, TNM score, histologic grade, primary tumor site, liver metastases, lung metastases, bone metastases, and number of metastatic sites.

### 2.3. Statistical Analysis

The databases used were downloaded using the SEERStat v8.4.2 software and exported to STATA v18.0. Descriptive statistics were used to summarize the general characteristics of the study population, which included 24,703 adults diagnosed with metastatic CRC. A multivariate logistic regression model was conducted to assess predictors of brain metastasis at initial stage IV colorectal cancer diagnosis; this method was chosen as it allows for the adjustment of multiple confounding variables simultaneously. Overall survival (OS) was calculated by using the Kaplan–Meier method, and the log rank test was used to compare differences between groups. Furthermore, a Cox proportional hazards regression model was used to assess associations between exposure variables and all-cause mortality. The multivariate model included adjustment variables with *p* < 0.05 using the backward selection method. However, race and sex were included in our multivariate analysis, although they were not statistically significant in our univariate analysis, to ensure that our analyses were robust, unbiased, and clinically meaningful. All *p* values < 0.05 were considered statistically significant.

## 3. Results

A total of 24,703 patients with metastatic colorectal cancer (mCRC) were included in the study. Among them, 228 patients (0.92%) had brain metastasis (BM) at the time of diagnosis, while 24,475 patients (99.1%) had metastases at other sites. Within patients with BM, 10.53% had early-onset mCRC (<50 years) compared to 89.57% with average-onset mCRC (≥50 years) (*p* = 0.004). Most patients with BM were male (52.32%, *p* = 0.693) and white (82.89%, *p* = 0.071) and had adenocarcinoma as the histologic subtype (88.60%, *p* = 0.023), a T3 score (46.93%, *p* < 0.001), an N2 score (43.42%, *p* = 0.04), and a moderately differentiated histologic grade (57.89%, *p* = 0.154). Additionally, most patients had their primary tumor located in the left colon (35.52%, *p* = 0.294) and metastasis to three sites or more (77.19%, *p* < 0.001). More details about the general characteristics according to the presence of BM at the time of diagnosis of mCRC can be found in Table 1.

Factors associated with the presence of BM at diagnosis in adults with mCRC are presented in Table 2. Average-onset mCRC was associated with an increased risk of presenting with BM (OR: 1.81, 95% CI: 1.18–2.78, *p* = 0.006) when compared to early-onset mCRC, as was having three or more sites of metastasis (OR: 6.71, 95% CI: 4.52–9.94, *p* < 0.001). In contrast, mucinous adenocarcinoma as the histological subtype was associated with a decreased risk of presenting with BM at the time of the initial CRC diagnosis (OR: 0.48, 95% CI: 0.24–0.98, *p* = 0.043).

**Figure 1:**
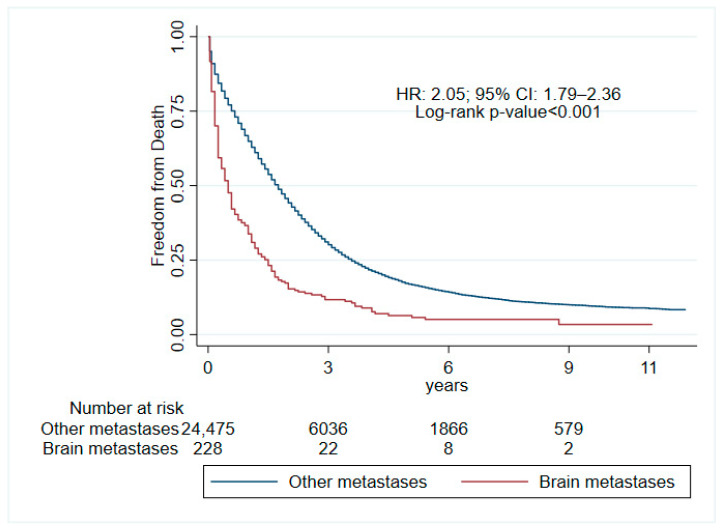
Overall survival of individuals with colorectal cancer and brain metastases compared to other metastatic sites at diagnosis.

Table 3 shows factors associated with all-cause mortality in adults with mCRC and BM at diagnosis. It was found that Asians/Pacific Islanders (HR: 1.83, 95% CI: 1.01–3.33, *p* = 0.045) and American Indians/Alaska Natives (HR: 4.79, 95% CI: 1.15–19.97, *p* = 0.032) had higher mortality compared to the white population. However, these results should be interpreted with caution due to the small sample size of American Indians/Alaska Natives (*n* = 2). Surgical treatment was associated with decreased mortality (HR: 0.49, 95% CI: 0.33–0.72, *p* < 0.001), as was chemotherapy (HR: 0.35, 95% CI: 0.26–0.48, *p* < 0.001). The Kaplan–Meier survival curve (Figure 1) comparing mortality rates between patients with brain metastases and those without reveals a statistically significant difference (*p* < 0.001). This demonstrates that patients with mCRC who present with brain metastases have significantly poorer overall survival compared to those without brain metastases. The median OS in patients with BM was 6 months compared to 21 months in patients without BM (*p* < 0.001).

## 4. Discussion

Brain metastasis from colorectal cancer has been well-documented in the past, with prevalence varying across studies [11,12]. This variability could be attributed to small sample sizes in the studies. A systematic review reported that the incidence of brain metastases from CRC is between 0.6% and 3.2%, identifying risk factors, such as young age, lung metastases, rectal primary, and KRAS mutation [13]. Additionally, increased levels of CEA at diagnosis have been associated with brain metastases [14]. To our knowledge, this is one of the most recent population-based studies providing contemporary insights into BM in metastatic colorectal cancer (mCRC) [15]. Our study highlights several important aspects regarding brain metastasis in patients with metastatic colorectal cancer. The overall prevalence of BM in our cohort was 0.90%, aligning with multiple studies [2,3,6,11,15]. 

We found that patients with average-onset mCRC are 84% more likely to present with brain metastases at diagnosis when compared to early-onset mCRC. The lower risk of brain metastasis observed in early-onset mCRC (<50 years) compared to average-onset mCRC (≥50 years) is particularly noteworthy, which may suggest underlying biological or molecular distinctions between early-onset and average-onset mCRC. Molecular distinctions between the two have been shown, with a study indicating a KRAS mutation rate of 1% in early-onset CRC compared to 32% in average-onset CRC [16]. This mutation was previously associated with an increased risk of BM in mCRC [4,17]. Furthermore, we found that having three or more sites of metastasis had an OR of 6.71 for presenting with BM when compared to having only one site, which has been shown in prior studies [3,4,11,15,17]. 

Additionally, we found that mucinous carcinoma is associated with a 52% lower risk of brain metastasis compared to adenocarcinoma of the colon. This observation has not been reported in the previous literature. Mucinous carcinoma has been shown to result in metastatic disease more frequently and to involve multiple metastatic sites compared to adenocarcinoma patients. However, previous studies have not identified a statistically significant difference in the risk of brain metastasis among different histological types [18]. This finding suggests that while mucinous carcinoma may lead to extensive metastatic spread, it appears to have a lower propensity for metastasizing to the brain specifically. Further research is needed to understand the underlying mechanisms and potential clinical implications of this distinction.

The overall survival (OS) indicates that the presence of brain metastases (BMs) in metastatic colorectal cancer (mCRC) doubles the mortality rate, leading to a worse prognosis. It has been found that 33% of patients with colorectal cancer (CRC) will develop metastases at presentation or during follow-up [19], and that the 5-year relative OS for patients with mCRC is approximately 15% [20]. The OS for BM in mCRC reported in other studies shows an average of 4 months (ranging from 1 to 13 months) [21]. Other research reports a median OS of up to 7 months, with one-year and two-year OS rates of 19.05% and 9.52%, respectively [22]. Similarly, our study found that the median OS in patients with BM was 6 months compared to 21 months in patients without BM (*p* < 0.001). These findings underscore the critical need for early detection and targeted treatment strategies for BM in mCRC patients. The stark difference in OS between patients with and without BM highlights the aggressive nature of brain metastases and their impact on patient outcomes, hence the importance of prompt diagnosis and treatment in these patients. 

Furthermore, the use of unimodal treatment has an average OS of less than 4 months, while multimodal treatments can increase it to up to 11 months [7]. Given that the blood–brain barrier limits the effectiveness of systemic chemotherapy, local treatment options, such as stereotactic radiosurgery (SRS), are necessary. In recent years, the utility of immunotherapy has been demonstrated in various studies in patients with brain metastases in different types of cancer, such as melanoma, breast cancer, and lung cancer [23,24].

Currently, guidelines for routine screening for brain metastases in CRC patients are not well-established [3], partly due to the relatively low incidence of BM in this population. However, our findings suggest that specific high-risk groups, such as patients with average-onset mCRC and those with multiple metastatic sites, might benefit from more vigilant surveillance for brain metastases. Incorporating targeted screening protocols for these high-risk populations could potentially lead to earlier detection and improved management of brain metastases, ultimately enhancing patient outcomes. Further studies are warranted to develop and validate such screening guidelines, thus ensuring they are both cost-effective and clinically beneficial.

This study has several limitations that should be acknowledged. First, as with any retrospective study, the ability to establish causality is inherently limited. The data were extracted from the SEER database, which, while comprehensive, may not capture all relevant clinical variables, such as neurological symptoms at the time of diagnosis. This omission could lead to residual confounding, as unmeasured factors might influence the observed associations. Another limitation is the potential for selection bias, as our analysis was restricted to patients with documented brain metastases (BMs) at the time of metastatic colorectal cancer (mCRC) diagnosis. This approach excludes patients who may develop BM later (metachronous BM), potentially limiting the generalizability of our findings. Additionally, some subgroups within our study population, such as American Indians/Alaska Natives, had small sample sizes, which hinders the extrapolation of these findings to the broader population.

Furthermore, this study lacks epidemiological data on KRAS mutations within the population, an important factor in understanding the genetic underpinnings of BM in mCRC. The limited availability of information and clinical trials specific to the management of BM in CRC further constrains the ability to compare our findings with established management guidelines. Despite these limitations, this study provides a valuable characterization and evaluation of the overall survival (OS) of patients with BM in mCRC, offering insights that could inform future research and clinical practice.

Building on the findings of this study, several areas for future research warrant attention. First, prospective studies are needed to validate the clinical predictors of brain metastasis (BM) identified in this study. Such studies could help establish more definitive causal relationships and provide a basis for developing risk-stratification tools for patients with metastatic colorectal cancer (mCRC). Future research should also focus on the inclusion of genetic and molecular data, particularly concerning KRAS and other relevant mutations, to better understand the genetic predisposition to BM in CRC patients. Integrating genomic information with clinical predictors could lead to the development of personalized screening protocols, enabling earlier detection and intervention.

Given the poor prognosis associated with BM in mCRC, studies exploring novel therapeutic approaches, such as targeted therapies or combination regimens that can penetrate the blood–brain barrier, are crucial. Additionally, clinical trials designed specifically for patients with CRC and BM are needed to evaluate the efficacy of these treatments and to establish evidence-based guidelines.

Lastly, research should also explore the disparities in BM incidence and outcomes among different racial and ethnic groups. Understanding these disparities could inform more equitable treatment approaches and potentially guide revisions to clinical guidelines to ensure they are inclusive of diverse patient populations.

## 5. Conclusions

Brain metastases (BMs) in metastatic colorectal cancer (mCRC) are typically detected in advanced and disseminated stages, resulting in poor prognosis and low overall survival (OS). Currently, there are no guidelines recommending routine screening for brain metastases in colorectal cancer patients. Our study highlights the critical factors associated with the presence of BM in mCRC patients and emphasizes the poor OS. Novel treatments, such as immune checkpoint inhibitors, have shown promising results in treating brain metastases in various cancers, underscoring the importance of including patients with brain metastases in clinical trials. These findings highlight the need for greater clinical awareness, early detection, and personalized therapeutic strategies to improve outcomes, treatment, and survival in this high-risk population.

## Figures and Tables

**Table 1:** General characteristics according to the presence of brain metastasis at diagnosis in adults with metastatic colorectal cancer.

Characteristics	All Sample (*n* = 24,703)		Brain Metastases (*n* = 228)		Other Metastases (*n* = 24,475)		*p*-Value
	*n*	%	*n*	%	*n*	%	
Age (years)							0.004 ^†^
<50	4387	17.76	24	10.53	4363	17.83	
≥50	20,316	82.24	204	89.57	20,112	82.17	
Mean ± SD	62.51 ± 13.74		62.49 ± 13.75		64.46 ± 12.61		0.032 ^‡^
Sex							0.693 ^†^
Female	11,706	47.39	111	48.68	11,595	47.37	
Male	12,997	52.61	117	51.32	12,880	52.63	
Race							0.071 ^†^
White	18,664	75.55	189	82.89	18,475	75.49	
Black	3538	14.32	24	10.53	3514	14.35	
Asian/Pacific Islander	2272	9.20	13	5.70	2259	9.23	
American Indian/Alaska Native	229	0.93	2	0.88	227	0.93	
Histologic subtype							0.023 ^†^
Adenocarcinoma	21,163	87.49	202	88.60	21,411	87.48	
Mucinous adenocarcinoma	1876	7.59	8	3.51	1868	7.63	
Large-cell neuroendocrine carcinoma	529	2.14	7	3.07	522	2.13	
Others	685	2.78	11	4.82	674	2.75	
Tumor size (mm)							0.246 ^¥^
Median (IQR)	51 (40–70)		55 (40–70)		51 (40–70)		
T score							<0.001 ^†^
T1	1055	4.27	15	6.58	1040	4.25	
T2	690	2.79	16	7.02	674	2.75	
T3	12,097	48.97	107	46.93	11,990	48.99	
T4	10,861	43.97	90	39.47	10,771	44.01	
N score							0.048 ^†^
N0	4556	18.44	55	24.12	4501	18.39	
N1	9454	38.27	74	32.46	9380	38.32	
N2	10,693	43.29	99	43.42	10,594	43.28	
Histologic grade							0.154 ^†^
Well-differentiated	1214	4.91	12	5.26	1202	4.91	
Moderately differentiated	15,980	64.69	132	57.89	15,848	64.75	
Poorly differentiated	6340	25.67	69	30.26	6271	25.62	
Undifferentiated	1169	4.73	15	6.59	1154	4.72	
Primary tumor site							0.294 ^†^
Right colon	4240	17.16	49	21.49	4191	17.12	
Left colon	10,017	40.55	81	35.53	9936	40.60	
Transverse colon	1797	7.27	14	6.14	1783	7.28	
Cecum	5416	21.92	50	21.93	5366	21.92	
Rectum	3233	13.09	34	14.91	3199	13.08	
Brain metastases							
No/unknown	24,475	99.08	-	-	-	-	
Yes	228	0.92	-	-	-	-	
Liver metastases							<0.001 ^†^
No/unknown	7474	30.26	122	53.51	7352	30.04	
Yes	17,229	69.74	106	46.49	17,123	69.96	
Lung metastases							<0.001 ^†^
No/unknown	20,063	81.22	139	60.96	19,924	81.41	
Yes	4640	18.78	89	39.04	4551	18.59	
Bone metastases							<0.001 ^†^
No/unknown	23,859	96.58	185	81.14	23,674	96.73	
Yes	844	3.42	43	18.86	801	3.27	
Number of metastatic sites							<0.001 ^†^
Only one site	21,940	88.82	176	77.19	21,764	88.92	
Two sites	2088	8.45	20	8.77	2068	8.45	
Three sites or more	675	2.73	32	14.04	643	2.63	
Vital Status							<0.001 ^†^
Alive	5546	22.45	23	10.09	5523	22.57	
Died	19,157	77.55	205	89.91	18,952	77.43	
Follow-up (years)							<0.001 ^¥^
Median (IQR)	1.42 (0.5–2.92)		0.46 (0.17–1.25)		1.42 (0.5–2.92		

SD: standard deviation; IQR: interquartile range. ^†^ Chi-squared test; ^‡^ T Student test; ^¥^ U Mann–Whitney test.

**Table 2:** Factors associated with the presence of brain metastasis at diagnosis in adults with metastatic colorectal cancer.

Exposure	Crude Model ^a^			Adjusted Model ^a,b^		
	OR	95% CI	*p*-Value	OR	95% CI	*p*-Value
Age (years)						
<50	Ref.	-	-	Ref.	-	-
≥50	1.84	1.21–2.82	0.005	1.81	1.18–2.78	0.006
Sex						
Female	Ref.	-	-	Ref.	-	-
Male	0.95	0.73–1.23	0.694	0.92	0.71–1.20	0.536
Race						
White	Ref.	-	-	Ref.	-	-
Black	0.67	0.44–1.02	0.063	0.66	0.43–1.01	0.054
Asian/Pacific Islander	0.56	0.32–0.99	0.045	0.58	0.33–1.03	0.060
American Indian/Alaska Native	0.86	0.21–3.49	0.834	0.82	0.20–3.36	0.786
Histologic subtype						
Adenocarcinoma	Ref.	-	-	Ref.	-	-
Mucinous adenocarcinoma	0.45	0.22–0.92	0.029	0.48	0.24–0.98	0.043
Large-cell neuroendocrine carcinoma	1.42	0.67–3.03	0.364	1.24	0.57–2.70	0.583
Others	1.73	0.94–3.19	0.079	1.79	0.97–3.33	0.065
T score						
T1	Ref.	-	-	Not evaluated ^†^		
T2	1.65	0.81–3.35	0.170			
T3	0.62	0.36–1.07	0.084			
T4	0.58	0.33–1.00	0.052			
N score						
N0	Ref.	-	-	Ref.	-	-
N1	0.65	0.45–0.92	0.014	0.69	0.48–1.01	0.051
N2	0.76	0.55–1.06	0.113	0.81	0.57–1.16	0.259
Histologic grade						
Well-differentiated	Ref.	-	-	Not evaluated ^‡^		
Moderately differentiated	0.83	0.46–1.51	0.550			
Poorly differentiated	1.10	0.60–2.04	0.757			
Undifferentiated	1.31	0.61–2.79	0.498			
Number of metastatic sites						
Only one site	Ref.	-	-	Ref.	-	-
Two sites	1.20	0.75–1.90	0.450	1.27	0.79–2.03	0.316
Three sites or more	6.15	4.19–9.04	<0.001	6.71	4.52–9.94	<0.001

OR: odds ratio; 95% CI: 95% confidence interval. ^a^ Logistic regression model. ^b^ Adjusted for age, sex, race, histologic subtype, N score, and number of metastatic sites. ^†^ Variables that did not enter the adjusted regression model because they presented collinearity with other variables. ^‡^ Variables that did not enter the adjusted regression model because they showed a *p*-value > 0.05 in the crude regression model.

**Table 3:** Factors associated with all-cause mortality in adults with colorectal cancer and brain metastasis at diagnosis.

Exposure	Crude Model ^a^			Adjusted Model ^a,b^		
	HR	95% CI	*p*-Value	HR	95% CI	*p*-Value
Age (years)						
<50	Ref.	-	-	Ref.	-	-
≥50	1.58	1.01–2.46	0.045	1.36	0.84–2.20	0.204
Sex						
Female	Ref.	-	-	Not evaluated ^†^		
Male	0.96	0.73–1.26	0.771			
Race						
White	Ref.	-	-	Ref.	-	-
Black	1.11	0.71–1.74	0.641	1.30	0.82–2.06	0.256
Asian/Pacific Islander	1.62	0.92–2.86	0.095	1.83	1.01–3.33	0.045
American Indian/Alaska Native	3.00	0.74–12.18	0.120	4.79	1.15–19.97	0.032
Histologic subtype				Not evaluated ^†^		
Adenocarcinoma	Ref.	-	-			
Mucinous adenocarcinoma	0.89	0.44–1.82	0.751			
Large-cell neuroendocrine carcinoma	1.35	0.63–2.88	0.434			
Others	1.01	0.53–1.91	0.985			
T score				Not evaluated ^†^		
T1	Ref.	-	-			
T2	0.96	0.45–2.05	0.913			
T3	0.74	0.40–1.35	0.324			
T4	0.86	0.47–1.59	0.641			
N score						
N0	Ref.	-	-	Ref.	-	-
N1	0.97	0.67–1.42	0.896	1.26	0.84–1.88	0.255
N2	1.11	0.78–1.58	0.557	1.26	0.85–1.86	0.250
Histologic grade						
Well-differentiated	Ref.	-	-	Ref.	-	-
Moderately differentiated	1.48	0.75–2.92	0.258	1.11	0.55–2.25	0.770
Poorly differentiated	1.74	0.87–3.50	0.120	1.40	0.67–2.88	0.368
Undifferentiated	1.54	0.67–3.57	0.312	1.54	0.63–3.74	0.339
Surgical treatment						
No/unknown	Ref.	-	-	Ref.	-	-
Yes	0.67	0.47–0.96	0.028	0.49	0.33–0.72	<0.001
Radiotherapy						
No/unknown	Ref.	-	-	Ref.	-	-
Yes	0.72	0.54–0.94	0.018	0.90	0.67–1.21	0.491
Chemotherapy						
No/unknown	Ref.	-	-	Ref.	-	-
Yes	0.35	0.26–0.47	<0.001	0.35	0.26–0.48	<0.001

HR: hazard ratio; 95% CI: 95% confidence interval. ^a^ Cox regression model. ^b^ Adjusted for age, race, N score, histologic grade, surgical treatment, radiotherapy, and chemotherapy. ^†^ Variables that did not enter the adjusted regression model because they showed a *p*-value > 0.05 in the crude regression model.

## Data Availability

The data presented in this study are available upon request from the corresponding author.
